# Parsing the functional specificity of Siderocalin/Lipocalin 2/NGAL for siderophores and related small-molecule ligands

**DOI:** 10.1016/j.yjsbx.2019.100008

**Published:** 2019-05-30

**Authors:** Matthew C. Clifton, Peter B. Rupert, Trisha M. Hoette, Kenneth N. Raymond, Rebecca J. Abergel, Roland K. Strong

**Affiliations:** a Division of Basic Science, Fred Hutchinson Cancer Research Center, Seattle, WA 98109, USA; b Seattle Structural Genomics Center for Infectious Disease, Seattle, WA, USA; c Department of Chemistry, University of California, Berkeley, CA 94720-1460, USA

**Keywords:** AEB, aerobactin, ABC, ATP‐binding cassette, AU, crystallographic asymmetric unit, BOCT, brain-type organic cation receptor, c-di-GMP, cyclic diguanylate monophosphate, CAM, catechol, CMB, carboxymycobactin, DHBA, dihydroxybenzoic acid, ENT, enterobactin or enterochelin, FQ, fluorescence quenching, HOPO, hydroxypyridinone, NGAL, Neutrophil Gelatinase Associated Lipocalin, NE, norepinephrine, PBP, bacterial periplasmic binding protein, PDB, Research Collaboratory for Structural Biology Protein Databank, PCH, pyochelin, PVD, pyoverdine, SBP, bacterial membrane-associated, substrate-binding protein, SCH, schizokinen, Scn, Siderocalin, Antimicrobial responses, Ferric enterobactin/enterochelin, Bacterial substrate binding proteins, X-ray crystallography

## Abstract

Siderocalin/Lipocalin 2/Neutrophil Gelatinase Associated Lipocalin/24p3 is an innate immune system protein with bacteriostatic activity, acting by tightly binding and sequestering diverse catecholate and mixed-type ferric siderophores from enteric bacteria and mycobacteria. Bacterial virulence achieved through siderophore modifications, or utilization of alternate siderophores, can be explained by evasion of Siderocalin binding. Siderocalin has also been implicated in a wide variety of disease processes, though often in seemingly contradictory ways, and has been proposed to bind to a broader array of ligands beyond siderophores. Using structural, directed mutational, and binding studies, we have sought to rigorously test, and fully elucidate, the Siderocalin recognition mechanism. Several proposed ligands fail to meet rigorous binding criteria, including the bacterial siderophore pyochelin, the iron-chelating catecholamine hormone norepinephrine, and the bacterial second messenger cyclic diguanylate monophosphate. While possessing a remarkably rigid structure, in principle simplifying analyses of ligand recognition, understanding Scn recognition is complicated by the observed conformational and stoichiometric plasticity, and instability, of its *bona fide* siderophore ligands. Since the role of Siderocalin at the early host/pathogen interface is to compete for bacterial ferric siderophores, we also analyzed how bacterial siderophore binding proteins and enzymes alternately recognize siderophores that efficiently bind to, or evade, Siderocalin sequestration – including determining the crystal structure of *Bacillus cereus* YfiY bound to schizokinen. These studies combine to refine the potential physiological functions of Siderocalin by defining its multiplexed recognition mechanism.

## Highlights

• Ligand recognition by antibacterial Siderocalin controls the competition for iron during infection.
• We determined nine crystal structures of Siderocalin mutants with ligands.
• We determined three candidate ligands did not bind.
• We determined the crystal structure of SBP YfiY.
• Multiplexed specificity of Siderocalin was determined.

## Graphical abstract

**Figure:**
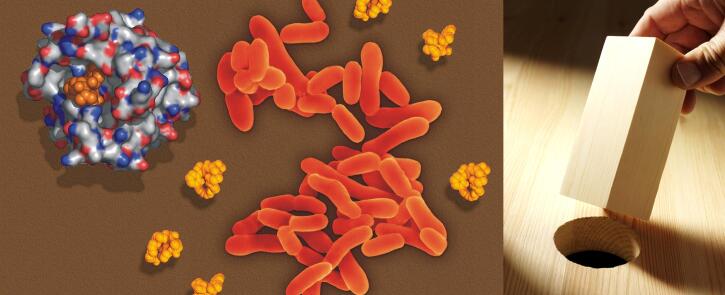

## 1 Introduction

The glycoprotein Siderocalin (Scn)/Lipocalin 2/Neutrophil Gelatinase Associated Lipocalin (NGAL)/24p3 (the *LCN2* chromosomal location in mice) was discovered as a component of human neutrophil granules as a monomer, disulfide-linked homodimer, and disulfide-linked heterodimer with Matrix Metalloproteinase 9/Gelatinase B (Correnti and Strong, 2012, Xiao et al., 2017). Subsequent studies reported Scn expression in a wide variety of organs, cell types, and tissues, including kidney, liver, uterus, leukocytes, glial cells, adipocytes, chondrocytes, keratinocytes, and epithelial cells (Moschen et al., 2017, Song and Kim, 2018). Scn has been implicated, though often in contradictory ways, in many disease processes, including bacterial infections, gut microbiota homeostasis, inflammatory bowel disease, psoriasis, obesity, insulin resistance, fatty liver disease, atherosclerosis, Alzheimer's disease and other neurodegenerative disorders, metabolic syndrome, renal disorders, and a wide range of cancers (Bauvois and Susin, 2018, Correnti and Strong, 2012, Moschen et al., 2017, Song and Kim, 2018, Xiao et al., 2017). Scn in serum and urine is also a useful biomarker of human inflammatory diseases, including acute kidney injury and chronic kidney disease.

**Fig. 1:**
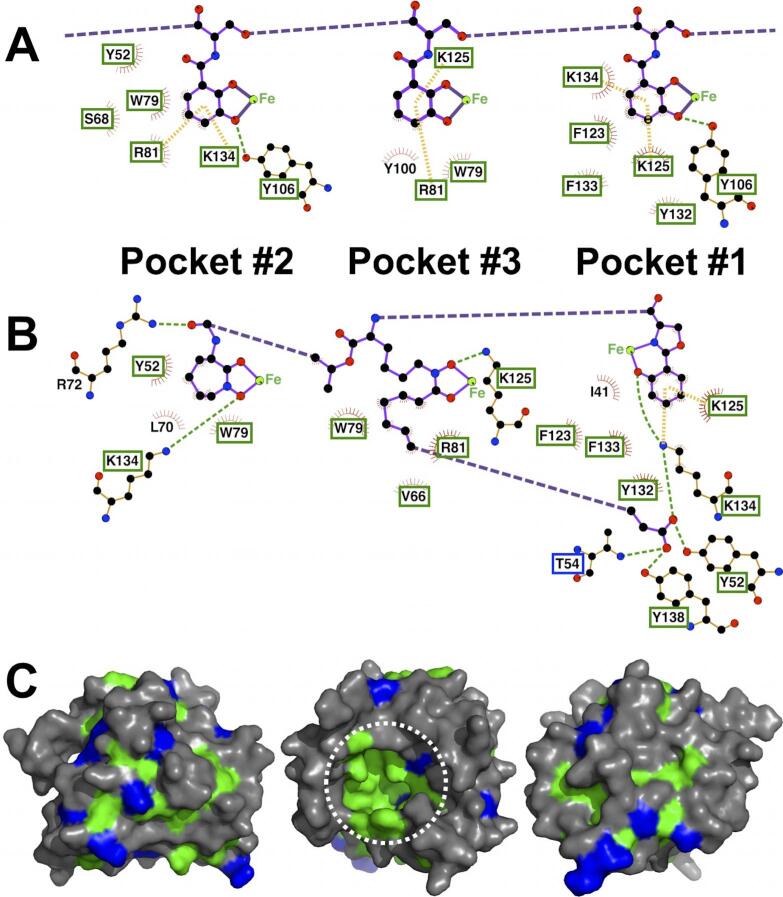
The Scn calyx is highly conserved across orthologs. Exploded LIGPLOT (Wallace et al., 1995) schematics of the interactions between (A) Scn and Fe-ENT, and (B) Scn and Fe-CMB, highlight the ligand/protein contacts in the calyx. Ligand covalent bonds are colored purple (exploded bonds are dashed), protein covalent bonds are colored orange, hydrogen bonds are indicated with dashed green lines, and cation-π interactions are shown as dashed yellow bars. Atoms are colored by type (C, black; O, red; N, blue, Fe, green). Van der Waals contacts are indicated by red sunbursts. Ligand-contacting residues completely conserved across 18 vertebrate Scn sequences (Correnti and Strong, 2013) have labels boxed in green. T54, substituted by serine in the *O. cuniculus* Scn sequence but otherwise conserved, is boxed in blue. (C) Three views of the molecular surface of Scn, rotated by 60° around the vertical axis relative to one another, are colored by conservation: residues completely conserved across 18 vertebrate Scn sequence are colored green, and residues conservatively substituted (V/I/L, T/S, T/I, T/V, E/D, D/N, F/Y, three or fewer substitutions among the 18 sequences) are colored blue. The calyx is circled by a white dashed line. (For interpretation of the references to color in this figure legend, the reader is referred to the web version of this article.)

Lipocalins as a family are secreted proteins which generally bind to and transport small hydrophobic molecules, such as steroids, bilins, retinoids, and lipids, with examples found in bacteria, plants, invertebrates, and vertebrates (Åkerstrom et al., 2000). Despite limited sequence similarity beyond minimal fold-defining motifs, lipocalins, including Scn (Coles et al., 1999, Goetz et al., 2000), display a common structural architecture: an eight-stranded antiparallel β-barrel enclosing a cup-shaped ligand binding site, or calyx. Sequence gazing (Correnti and Strong, 2013) identified Scn orthologs from rodents, ruminants, and canines, predicted to have a conserved ligand specificity based on near total conservation of calyx-lining residues (Fig. 1A–C), which was confirmed for murine Scn (Correnti et al., 2012).

**Fig. 2:**
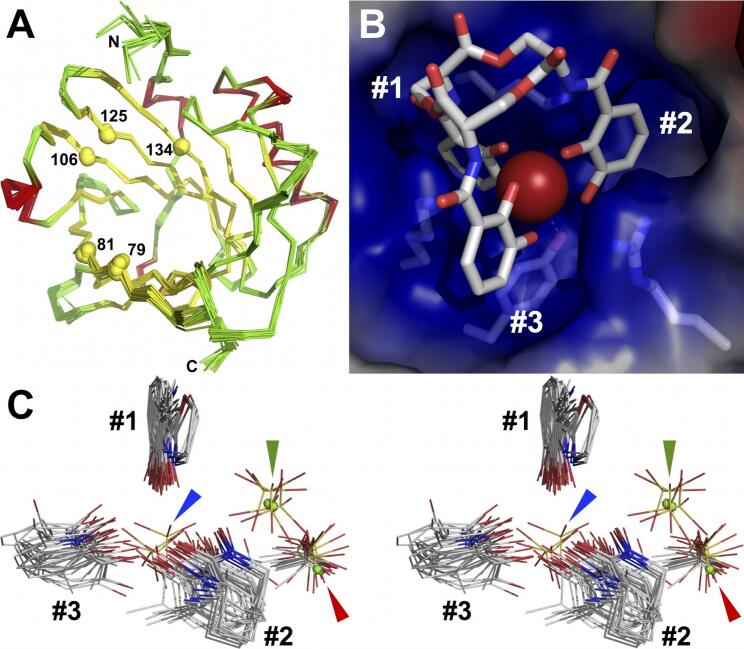
The rigid Scn calyx highly constrains ligand recognition. (A) A superposition of 36 independent views of the crystal structure of human Scn, assembled from multiple *apo* and ligand-bound structures, in backbone representations colored by secondary structure (helix: red, strand: yellow, coil: green), highlights the overall rigidity of the Scn fold. The Cα atoms of key ligand-contacting residues are shown as spheres and numbered, and N- and C-termini are labeled. The superposition includes the mutant Scn structures discussed in this report. (B) A detailed view of the modeled structure of intact Fe-ENT bound in the Scn calyx is shown, based on crystal structures of Scn bound with partially degraded Fe-ENT. Scn is shown as a semi-transparent molecular surface colored by charge, with the side-chains of key ligand-contacting residues shown in a licorice-stick representation. The three pockets in the trilobate calyx are numbered. (C) A stereoview of the superposition of key ligand substituents bound in calyx pockets from 44 independent views of Scn/ligand complex crystal structures reveals calyx pocket specificities. CAM and hydroxyphenol oxazoline substituents are tightly-constrained in Pocket #1. CAM, HOPO, linear hydroxamate, and cyclic hydroxamate substituents are more loosely bound in Pockets #2 and #3. Sulfate and chloride ions, and terminal CMB carboxylates, bind in the deepest chamber of Pocket #2 (red arrow), while sulfates and chlorides bind between Pockets #1 and #2 (green arrow) in structures with ligands with reduced overall negative charge. Sulfate ions occupy the center of the calyx, near to the position of chelated metals in siderophore complexes, in *apo* structures and the phenylurea complex structure (blue arrow). (For interpretation of the references to color in this figure legend, the reader is referred to the web version of this article.)

Lipocalin function can be understood in terms of the specific ligands bound, and subsequent interactions with specific, cell-surface receptors. Despite displaying an exceptionally rigid structure overall (Fig. 2A) and within its calyx (Allred et al., 2015), Scn has been reported to bind a dizzying array of polar or negatively-charged small-molecule ligands. These include: multiple families of natural siderophores and synthetic iron chelators (*tris ortho*-catechol (CAM) examples from enteric bacteria (Abergel et al., 2006a, Abergel et al., 2006b, Abergel et al., 2008, Allred et al., 2013, Doneanu et al., 2004, Goetz et al., 2002, Hoette et al., 2008) and mixed-type carboxymycobactins (CMBs) (Hoette et al., 2011, Holmes et al., 2005)); ferric complexes of simple *ortho*-CAMs (Bao et al., 2010, Barasch et al., 2016); the ferric complex of the neuroendocrine catecholamine hormone *L*-norepinephrine (Miethke and Skerra, 2010); ferric complexes of *meta*-CAMs (Devireddy et al., 2010); synthetic hydroxypyridinone (HOPO)-based lanthanoid and actinide chelators (Allred et al., 2015, Captain et al., 2016, Deblonde et al., 2017); and the bacterial second messenger cyclic diguanylate monophosphate (c-di-GMP; (Li et al., 2015)). Binding of siderophores can account for the well-established anti-bacterial activity of Scn (Flo et al., 2004, Moschen et al., 2017, Wilson et al., 2016, Xiao et al., 2017), sequestering iron needed by pathogens during infection, and may thereby have diverse physiological effects involving inflammation. Any effect on normal iron homeostasis could also affect a wide range of physiological processes, though such an effect would require an endogenous siderophore or equivalent iron chelating moiety, since Scn does not bind iron in isolation (Goetz et al., 2002). Scn has also been reported to bind to two different endogenous receptors: Megalin/low density lipoprotein-related protein 2 (Hvidberg et al., 2005); and brain-type organic cation receptor (BOCT)/SLC22A17/24p3R (Devireddy et al., 2005).

We have sought to understand in detail how Scn can tightly bind, often with sub-nanomolar equilibrium dissociation constants (*K_D_*s), such a wide range of chemically-distinct small-molecule ligands, and report here a series of binding and crystallographic analyses of selected compounds and Scn mutants, combining to reveal a remarkable, multiplexed recognition mechanism. Noting, however, that the identifications of BOCT as a candidate Scn receptor, and *meta*-CAMs as candidate endogenous ligands, have been directly challenged by subsequent studies (Cabedo Martinez et al., 2016, Correnti and Strong, 2012, Correnti et al., 2012), we also sought to rigorously test whether all reported ligands actually bind with functionally-relevant affinities, and found several discrepancies. Rigorous identification of Scn ligands, elucidation of Scn recognition mechanisms, and clearing “red herring” candidate ligands from consideration are important for determining the precise physiological role/s of Scn in health and disease. We also contrasted Scn with bacterial recognition of ferric siderophores, determining the crystal structure of the *Bacillus cereus* membrane-associated, substrate-binding protein (SBP) YfiY in complex with ferric schizokinen (SCH), an analog of aerobactin (AEB), a siderophore used by virulent bacteria to evade Scn-mediated iron blockade (Flo et al., 2004, Goetz et al., 2002, Sheldon and Heinrichs, 2015).

## 2 Results and discussion

### 2.1 Scn calyx pocket specificities

The Scn calyx is trilobate (Fig. 2B), with pockets accommodating the three 2,3-CAM rings of siderophores used by many enteric bacteria, e.g., enterobactin/enterochelin (ENT), or the hydroxyphenyl oxazoline, heterocyclic hydroxamate, and linear hydroxamate groups of CMBs (Fig. 1A and B) (Goetz et al., 2002, Holmes et al., 2005). Pocket #1 is the most constrained, tightly fitting phenyl groups substituted on the 1, 2, and/or 3 carbons, generating optimized van der Waals contacts, but the other two pockets are more open, more loosely binding a variety of ligand substituents (Fig. 2C). Pocket #2 is the deepest pocket in the calyx, with a compartment unfilled by *tris ortho*-CAM siderophores, readily apparent in Fig. 2B, but which accommodates the terminal carboxylate of ferric CMBs, or ions in other complexes. The calyx is strongly electropositive overall, due to the close arrangement of the side-chains of two lysines (K125, K134) and an arginine (R81), which complements the net negative charge of many ferric siderophores. These side-chains also generate circularly-permuted cation-π interactions with CAM substituents, which were previously concluded to predominate over electrostatic contributions to binding (Hoette et al., 2008). The cation-π interaction from R81 alternates with a ring-stacking interaction from a tryptophan (W79) in various structures. The side-chains of R81 and W79 are the most structurally-mobile elements in the calyx, adopting a range of different rotamers, or simply becoming disordered, to accommodate different ligands, or even between different molecules in the asymmetric units (AU) of Scn crystal structures. The only protein atom/s approaching the chelated iron in complexes with *tris*-CAM siderophores is the hydroxyl of Y106, underlying the bound ligand, but this group is typically not within van der Waals contact distance, at ≥3.8 Å away from the iron (Fig. 1A). The iron atom in ferric CMBs is held higher out in the calyx, yet further away from direct protein contacts. Minimizing geometry-dependent hydrogen bonds in the ENT complex (Fig. 1A) likely enables increased recognition degeneracy across CAM-based siderophores from enteric bacteria, broadening the Scn defense.

**Fig. 3:**
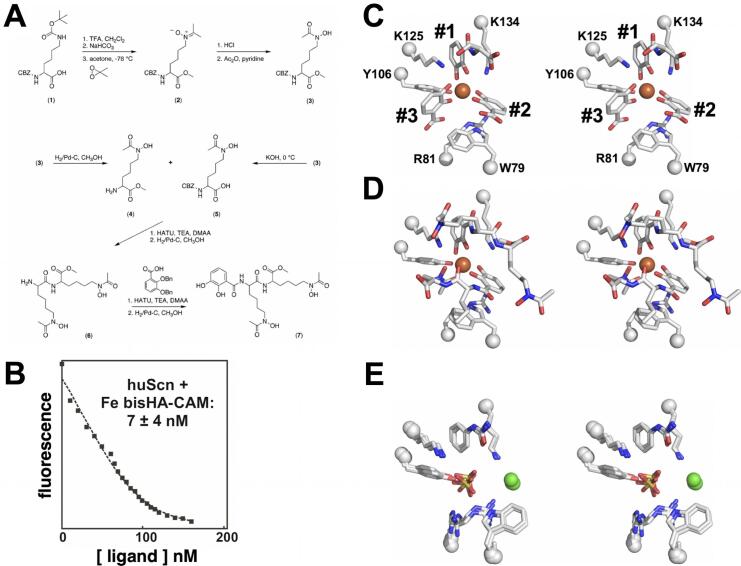
Identification of the Scn calyx pocket key for ligand recognition. (A) The stepwise synthesis of bisHA-CAM is detailed. (B) The binding of Fe-bisHA-CAM was quantitated by FQ as in (Abergel et al., 2006a, Abergel et al., 2006b, Goetz et al., 2002, Hoette et al., 2008, Miethke and Skerra, 2010): *K_D_* = 7 ± 4 nM. (C) For reference with subsequent structures, a stereoview of the binding of degraded Fe-ENT in the Scn calyx is detailed (compare with the modeled complex with intact Fe-ENT, Fig. 2B). The side-chains of key ligand-contacting residues and visualized ligand substituents are shown in a licorice-stick representation, colored by atom type (C, grey; N, blue; O, red; S, yellow; Fe, orange; Cl, green) and labeled, with Cα positions marked with spheres. Calyx pockets are numbered as in Fig. 1B. In this view, a DHBA-serine substituent occupies Pocket #1, and DHBA substituents occupy Pockets #2 and #3 (the DHBA group in Pocket #3 sits in an inverted orientation, carboxylate towards the protein, allowed by partial degradation of bound ENT). (D) The 1:2 Fe:bisHA-CAM complex is shown bound in the Scn calyx in the most-ordered molecule in the AU, in the same orientation and style as Fig. 3C. Two complete bisHA-CAM moieties are fully resolved and modeled. bisHA-CAM ligands are progressively less well ordered in the other two complexes in the crystal structure AU, but otherwise showed identical binding. (E) The superposition of all three molecules in the AU of the Scn/phenylurea complex structure (3TZS.pdb) are shown, in the same orientation and style as Fig. 3C, with the phenylurea ligands bound in Pocket #1. Note the centrally-bound sulfate ions and peripherally-bound chloride ions. (For interpretation of the references to color in this figure legend, the reader is referred to the web version of this article.)

The consensus of previous studies was that Pocket #1 was the key pocket for ligand recognition, often with the only ordered element of partially disordered or degraded ligands, though this supposition had not been confirmed by direct experiment. [While the modeled complex of intact Fe-ENT bound to Scn is shown in Fig. 2B, Fe-ENT invariably degrades in Scn complex crystals to dihydroxybenzoic acid (DHBA) and DHBA-serine, the result of hydrolysis of the ENT triserine trilactone backbone and amide linkages (Goetz et al., 2002).] To accomplish this, we synthesized a compound, bisHA-CAM, with a single CAM group linked to two linear hydroxamates (Fig. 3A). Structural analysis of the binding of this compound was intended to show which Scn calyx pocket had the strongest preference for an isolated, polarized, iron-coordinating CAM substituent. Using an established fluorescence quenching (FQ) binding assay (Abergel et al., 2006a, Goetz et al., 2002), we showed that ferric bisHA-CAM binds Scn with a low nanomolar *K_D_* within 20-fold of the previously-reported 0.4 nM *K_D_* for Fe-ENT (Abergel et al., 2006a, Abergel et al., 2008, Goetz et al., 2002) (Fig. 3B). Stoichiometric 1:1 ferric bisHA-CAM complexes were co-crystallized with the C87S mutation of human Scn, which prevents homodimerization (Goetz et al., 2002), which supported a structure determination by x-ray crystallography (d_min_ = 2.8 Å, Table 1). Compared to the crystal structure of degraded Fe-ENT bound to Scn (Fig. 3C), Fe-bisHA-CAM bound with the CAM moiety in an essentially identical orientation in Pocket #1 (Fig. 3D). However, quite unexpectedly, bisHA-CAM bound in a 2:1 complex with iron in Scn complexes, with CAM substituents from two bisHA-CAM molecules providing four iron ligands, and the hydroxamate from one bisHA-CAM completing hexavalent coordination of the iron atom. Two full bisHA-CAM molecules were visualized in the best-ordered molecule in the AU, showing that this compound had not degraded during crystallization. [The most commonly observed tetragonal crystal form, adopted by all the structures reported here, has three Scn molecules in the AU, typically displaying increasing static disorder.] We interpreted this result to show that Pocket #2 has a strong enough preference for catechol over linear hydroxamate to reapportion 1:1 bisHA-CAM:Fe complexes into 2:1 complexes during Scn complexation. [Reapportionment of iron/siderophore chelates in crystal structures has been observed before, for instance in the periplasmic binding protein (PBP) CeuE/MECAM complex structure (Muller et al., 2006).] However, unfortunately, this approach did not resolve the relative preference of Pockets #1 and #2 for catechol, only that both pockets have stronger preferences for catechols than Pocket #3.

**Table 1:** Crystallographic data collection and refinement statistics for Scn/model ligand complex structures.

Protein	human Scn	human Scn	human Scn	human Scn	human Scn
Mutations	C87S	C87S	C87S	C87S	C87S
Ligand	Fe-bisHA-CAM	Fe-tren(CAM)_2_ (1,2-HOPO)	Fe-tren(CAM) (1,2-HOPO)_2_	Eu-tren(CAM) (1,2-HOPO)_2_	*apo*-ENT
Accession Code	3HWE	3HWF	3HWG	3TF6	3K3L
Space group	P4_1_2_1_2	P4_1_2_1_2	P4_1_2_1_2	P4_1_2_1_2	P4_1_2_1_2

Cell dimensions					
a = b, c (Å)	115.43, 119.29	114.8, 118.35	114.08, 118.42	114.09, 118.19	114.20, 119.30
α = β = γ (°)	90	90	90	90	90
Resolution (Å)	50.0–2.80 (2.90–2.80)	50.0–3.20 (3.28–3.20)	50.0–2.19 (2.25–2.19)	50.0–2.35 (2.41–2.35)	50.0–2.62 (2.69–2.62)
*R*_merge_	0.078 (0.24)	0.135 (0.44)	0.094 (0.43)	0.068 (0.13)	0.057 (0.41)
I/σ(I)	12.6 (4.0)	22.2 (6.26)	24.3 (6.6)	5.13 (4.10)	50.3 (7.51)
Completeness (%)	97.2 (96.0)	99.7 (99.5)	99.8 (100)	99.6 (99.4)	100 (99.9)
Redundancy	5.5 (4.8)	13.6 (13.7)	10.5 (10.6)	14.8 (2.3)	12.9 (13.2)
Fe peak heights (σ)	18, 14, 17	8, 7, 5	7, 17, 18	*NA*	None observed
*R*_work_/*R*_free_	0.230/0.283	0.218/0.265	0.242/0.268	0.192/0.214	0.260/0.307

*No. atoms*					
Protein	4062	3920	4008	4077	3682
Heterogen	198	159	239	77	128
Waters	84	17	167	117	116
Wilson B (Å^2^)	40.7	46.4	37.7	38.5	45.4
Average B (Å^2^)	34.0	31.0	31.1	38.0	47.0

*R.m.s. deviations*					
Bond lengths (Å)	0.006	0.016	0.005	0.016	0.005
Bond angles (°)	1.08	2.95	1.04	1.54	0.91

Values in parentheses are for the highest-resolution shell; NA: not applicable.

Serendipitously, a structural genomics consortium (Myler et al., 2009) determined and deposited a relevant Scn co-crystal structure with phenylurea (Fig. 3E) as part of a fragment-screening effort. Phenylurea bound in Pocket #1 in an orientation superimposable on previous structures, as well as a secondary position on the surface of the protein distal to the calyx. Sulfate ions were observed in the center of the calyx, superimposable on iron positions in complex structures with ferric siderophores. This result showed the dominant preference of Pocket #1 for phenyl groups over other calyx pockets, at least for unpolarized ones, supporting the assignment of this as the key pocket for determining ligand binding. Prior studies had shown that single methyl adducts at the 4 or 5 positions could be tolerated by Scn on one or two CAM or HOPO groups, but not on all three (Abergel et al., 2006a, Hoette et al., 2008, Holmes et al., 2005). Combined with this assignment of the key pocket, and results of the structural analysis of pocket constraints (Fig. 2C), we concluded that Pocket #1 cannot tolerate adducts on the 4 or 5 positions and is very unlikely to tolerate adducts on the 6 position.

### 2.2 The effect of overall ligand charge on binding

**Fig. 4:**
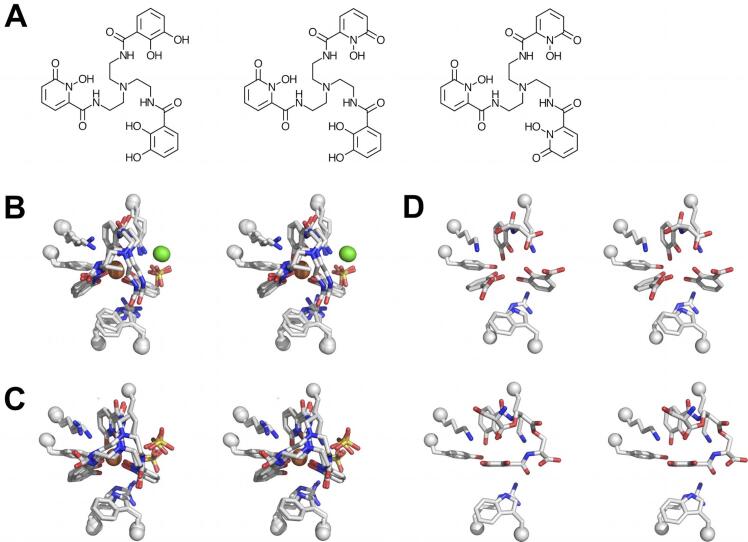
Effect of overall ligand charge on Scn binding. (A) Chemical structures of, left-to-right, tren(CAM)_2_(1,2-HOPO), tren(CAM)(1,2-HOPO)_2_, and tren(1,2-HOPO)_3_. Stereoviews of the superposition of the three (B) tren(CAM)_2_(1,2-HOPO)/Scn complexes, and (C) tren(CAM)(1,2-HOPO)_2_/Scn complexes, in the AU of their crystal structures, styled and oriented as in Fig. 3C. (D) Stereoviews of the best two ordered complexes of *apo*-ENT in the complex structure with Scn, styled and oriented as in Fig. 3C. ENT could not be resolved and confidently modeled in the third complex due to static disorder.

The net charge on Fe-ENT is −3, complementary to the strongly electropositive Scn calyx. We had previously used a series of isosteric ENT analogs (Fig. 4A), tren(CAM)_2_(1,2-HOPO), tren(CAM)(1,2-HOPO)_2_, and tren(1,2-HOPO)_3_, which have net charges of −2, −1, and 0 as complexes with iron, to isolate the effect of overall ligand charge on affinity (Hoette et al., 2008). The Scn *K_D_*s shifted from 0.4 nM for Fe-ENT, to 0.8 nM for Fe-tren(CAM)_2_(1,2-HOPO), to 43 nM for Fe-tren(CAM)(1,2-HOPO)_2_, to > 0.6 μM for Fe-tren(1,2-HOPO)_3_. We determined co-crystal structures with Fe-tren(CAM)_2_(1,2-HOPO) (d_min_ = 3.2 Å, Table 1, Fig. 4B) and Fe-tren(CAM)(1,2-HOPO)_2_ (d_min_ = 2.19 Å, Table 1, Fig. 4C), but co-crystallization with Fe-tren(1,2-HOPO)_3_ failed to yield interpretable diffraction data. The structures were notably similar, with all three ligands intact, well-resolved, and cleanly-interpretable in all three molecules in both respective crystal AUs. The binding of both analogs closely mimicked each other and the binding of Fe-ENT (Figs. 2B and 3C), validating the use of these nonhydrolyzable chelators as ENT surrogates. Differences in overall ligand charge were not accommodated by structural changes in the calyx, but by binding of negatively-charged counterions: well-resolved chloride and sulfate ions in the Fe-tren(CAM)_2_(1,2-HOPO) complex, and a pair of well-resolved sulfate ions in the Fe-tren(CAM)(1,2-HOPO)_2_ complex (both were crystallized using (NH_4_)_2_SO_4_ as the precipitant). We also determined the co-crystal structure with Eu-tren(CAM)(1,2-HOPO)_2_ (d_min_ = 2.35 Å, Table 1), though this structure recapitulates all the salient features of the iron complex, so is not detailed further.

In order to visualize binding of a neutrally-charged ligand, Scn was alternately co-crystallized with *apo*-ENT (d_min_ = 2.62 Å, Table 1, Fig. 4D, *K_D_* = 3.6 nM (Abergel et al., 2006a)). In the best ordered molecule in the AU, *apo*-ENT binding recapitulated Fe-ENT binding closely, including degradation into DHBA and DHBA-serine moieties. In the second molecule in the AU, a different degradation product was observed, two DHBA-serine groups coupled through a lactone linkage. One CAM substituent showed conserved interactions in Pocket #1, reinforcing the assignment of this pocket as “key”, but the second CAM substituent reoriented to a position overlapping that occupied by the iron atom, when present (Fig. 4D). The ligand in the third molecule in the AU could not be cleanly resolved and modeled. These results combine to show that overall ligand charge does not strongly affect the details of binding, which are likely driven by shape complementarity and cation-π interactions but serves to modulate affinity through overall Coulombic contributions.

### 2.3 How many mutations does it take to ablate Scn/ligand binding?

**Fig. 5:**
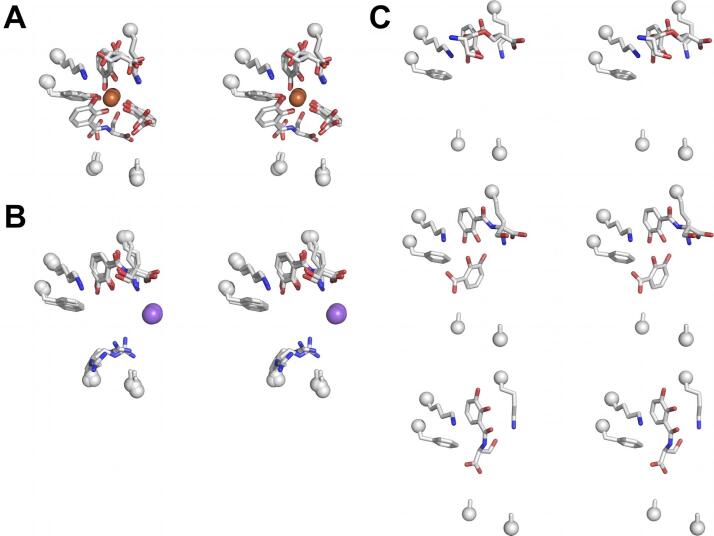
Effects of mutating distal calyx-lining residues on ligand recognition. Stereoviews of the superpositions of the three Fe-ENT complexes in the crystallographic AU of the (A) Scn(C87S/W79A/R81A) and (B) Scn(C87S/Y106) mutants, oriented and styled as in Fig. 3C. Even though W79 was not mutated in (B), its side-chain is disordered in this structure, and was modeled as alanine. (C) Separate stereoviews of the three Fe-ENT/Scn(C87S/W79A/R81A/Y106F) mutant complexes in the crystallographic AU.

The Scn calyx side-chains most closely contacting ligands are K125 and K134, together bracketing Pocket #1, W79 and R81, typically swapping rotamers to divide Pockets #2 and #3, and Y106, creating the central floor of the calyx under the chelated metal (Fig. 2B). We had previously determined Fe-ENT *K_D_*s and co-crystal structures for the Scn(C87S/W79A/R81A) and Scn(C87S/Y106F) mutants (Abergel et al., 2008). The W79A/R81A mutation reduced the affinity for Fe-ENT ∼175-fold, from 0.4 nM to 71 nM, and the Y106F mutation reduced the affinity ∼50-fold, from 0.4 nM to 20 nM. The structure of the Scn(C87S/W79A/R81A) mutant (Fig. 5A) was quite similar to that of wild-type Scn (Fig. 3C), with the affinity reduction readily accounted for simply by the loss of W79 and R81 contacts and interactions. The structure of the minimal Scn(C87S/Y106F) mutant, however, was dramatic and surprising, showing complete loss of bound iron and two of three CAM substituents from degraded ENT (Fig. 5B). This result remains difficult to reconcile, as the only ligand contact involving the hydroxyl of Y106 is a hydrogen bond to the 3-hydroxyl of the CAM substituent in Pocket #1 (Fig. 1A). In order to determine the effect of continued mutation on the overall structure of Scn and ligand binding, we produced the combination mutant Scn(C87S/W79A/R81A/Y106F) and determined its co-crystal structure (d_min_ = 2.3 Å, Table 2; Fig. 5C). Overall, this combination mutant showed ligand features similar in essential details to the Scn(C87S/Y106F) mutant. None of these mutations noticeably altered the Scn fold (Fig. 2A).

**Table 2:** Crystallographic data collection and refinement statistics for Scn mutants and candidate ligands.

Protein	human Scn	human Scn	human Scn	human Scn	human Scn	human Scn	human Scn
Mutations	C87S/W79A/R81A/Y106F	C87S/K125A	C87S/K134A	C87S/K125A/K134A	C87S	C87S	C87S
Ligand	Fe-ENT	Fe-ENT	Fe-ENT	Fe-ENT	(Fe-PCH)	(Fe-NE)	Fe-(NE) + DHBA
Accession Code	3T1D	3CMP	3I0A	3HWD	6O5D	–	–
Space group	P4_1_2_1_2	P4_1_2_1_2	P4_1_2_1_2	P4_1_2_1_2	P4_1_2_1_2	P4_1_2_1_2	P4_1_2_1_2

Cell dimensions							
a = b, c (Å)	114.75, 119.07	114.91, 118.83	114.25, 117.95	115.83, 119.32	114.8, 119.24	115.29, 118.31	114.97, 118.63
α = β = γ (°)	90	90	90	90	90	90	90
Resolution (Å)	50.0–2.30 (2.36–2.30)	50.0–2.80 (2.87–2.80)	50.0–2.60 (2.70–2.60)	50.0–2.95 (3.10–2.95)	50.0–2.40* (2.46–2.40)	50.0–2.40 (2.44–2.40)	50.0–2.25 (2.29–2.25)
*R*_merge_	0.072 (0.52)	0.077 (0.41)	0.123 (0.39)	0.084 (0.36)	NR (NR)*	0.077 (0.47)	0.074 (0.55)
I/σ(I)	10.5 (8.5)	26.0 (5.9)	12.4 (4.9)	25.3 (5.8)	5.78 (NR)*	49.5 (7.4)	56.3 (7.2)
Completeness (%)	96.9 (99.1)	99.0 (99.5)	99.9 (99.7)	91.0 (93.7)	95.7 (97.4)*	99.3 (100.0)	100 (100)
Redundancy	5.7 (4.8)	9.8 (9.9)	8.7 (8.6)	25.9 (6.2)	NR (8.4)*	8.0 (8.8)	14.1 (14.7)
Fe peak heights (A, B, C; σ)	None observed	11, 5, 13	16, 8, 16	None observed	None observed	None observed	10, 3, 10
*R*_work_/*R*_free_	0.189/0.228	0.248/0.304	0.255/0.300	0.257/0.303	0.235/0.253	–	–

*No. atoms*							
Protein	4130	3911	3902	3865	4026	–	–
Heterogen	114	161	145	7	37	–	–
Waters	259	93	100	51	73	–	–
Wilson B (Å^2^)	32.3	44.0	52.3	51.6	35.8	–	–
Average B (Å^2^)	32.0	39.0	46.0	43.0	27.6	–	–

*R.m.s. deviations*							
Bond lengths (Å)	0.018	0.005	0.006	0.005	0.010	–	–
Bond angles (°)	1.97	0.941	0.924	0.792	1.390	–	–

Values in parentheses are for the highest-resolution shell. *: From PDB entry 3U03. NR: Not reported.

**Fig. 6:**
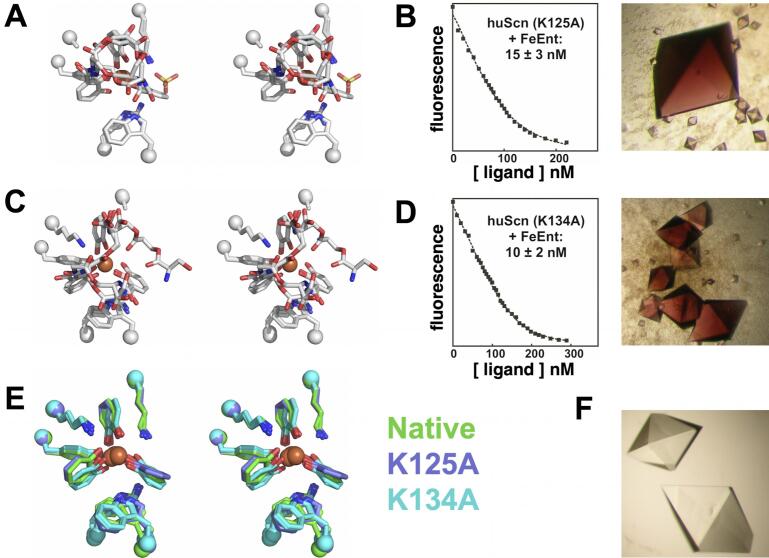
Effects of mutating proximal calyx-lining residues on ligand recognition. (A) Superpositions of the three Fe-ENT complexes in the crystallographic AU of the complex with the Scn(C87S/K125A) mutant are shown in a stereoview, oriented and styled as in Fig. 3C. (B) The FQ binding quantitation of the Fe-ENT/Scn(C87S/K125A) is shown, determined as in (Abergel et al., 2006a, Abergel et al., 2006b, Goetz et al., 2002, Hoette et al., 2008, Miethke and Skerra, 2010) (*K_D_* = 15 ± 3 nM), alongside a photograph of the complex crystals, which confirms binding qualitatively. (C) Superpositions of the three Fe-ENT complexes in the crystallographic AU of the complex with the Scn(C87S/K134A) mutant are shown in a stereoview. (D) The FQ binding quantitation of the Fe-ENT/Scn(C87S/K134A) is shown (*K_D_* = 10 ± 2 nM), alongside a photograph of the complex crystals. (E) Superpositions of the co-crystal structures of native, K125A, and K134A forms of Scn, colored as indicated, showing only the CAM rings of the bound ligands. (F) Crystals of Scn(C87S/K125A/K134A) grown in the presence of Fe-ENT. Lack of color demonstrated lack of ligand binding.

Growing somewhat frustrated with our inability to fully ablate ligand binding by even fairly extensive mutation, we focused on alternate mutations involving key Pocket #1: K125A, K134A, and the combination. Both lysine side-chains contribute bracketing Coulombic, cation-π, and hydrophobic interactions to the CAM group in this calyx pocket (Fig. 1A). We determined the Fe-ENT co-crystal structure of Scn(C87S/K125A) (d_min_ = 2.8 Å, Table 2; Fig. 6A) and its affinity by FQ (15 ± 3 nM; Fig. 6B), with binding qualitatively confirmed by co-crystallization (Fig. 6B), and the co-crystal structure of Scn(C87S/K134A) (d_min_ = 2.8 Å, Table 2; Fig. 6C) and its affinity (10 ± 2 nM; Fig. 6D), with binding also qualitatively confirmed by co-crystallization (Fig. 6D). The observed < 40-fold affinity reductions were surprising, given the seemingly crucial interactions contributed by these two side-chains bracketing the key pocket.

Examining their crystal structures, the two mutations had direct consequences for ligand stability, revealing nearly (K134A) or fully (K125A) intact ENT bound in the Scn calyx (Fig. 6A and B), likely the result of relaxation of the ENT triserine backbone into less hydrolysis-prone conformations. However, retention of more complete backbones through reduced hydrolysis generates additional protein contacts, likely buffering otherwise expected affinity reductions. A comparison of CAM ring positions (Fig. 6E) showed subtle rearrangements, particularly in Pocket #3, potentially permitting backbone relaxation, though in the absence of significant changes in Scn side-chain positions. The Y106 and lysine mutation results also suggested that Scn/Fe-ENT interactions have evolved to foster ENT hydrolysis while retaining degradation product binding, though the biological rationale for such a mechanism is unclear. Iron is released from ENT chelates in the cytoplasm of *E. coli* through the action of specific esterases (Schalk and Guillon, 2013), where iron affinity is reduced by converting hexadentate coordination to *tris* bidentate coordination, but the bacteriostatic activity of Scn requires retention of iron in ternary Scn-siderophore-iron complexes, to sequester it away from pathogens. Therefore, binding mechanisms that destabilize ENT, by fostering hydrolysis, would seem to be counterproductive. The Scn mechanism that fosters ENT hydrolysis is also unique, as ENT esterases utilize serine catalytic triads to enzymatically hydrolyze lactone backbone linkages, elements for which there is no analog in the Scn calyx.

The crystallographic analysis of the Scn(C87S/K125A/K134A) mutant (d_min_ = 2.95 Å, Table 2) finally achieved the desired result of fully ablating ligand binding, based on the absence of any ligand feature in the calyx, particularly bound iron, and qualitative co-crystallization binding trials (Fig. 6F), in the absence of any significant effect on the overall fold of Scn (Fig. 2A). Parallel efforts by Skerra and coworkers to “reprogram” Scn specificity through combinatorial mutagenesis to bind either ferric petrobactin, a virulence-associated siderophore of *Bacillus anthracis* (Dauner et al., 2018, Sheldon and Heinrichs, 2015), or lanthanoid/diethylenetriamine pentaacetate chelates (Kim et al., 2009), required ∼21 or at least 15 mutations, respectively, dramatically restructuring the calyx in the process. These results confirmed both the effort needed to alter the inherent ligand specificity of Scn, and the utility of the underlying fold for generating multiple, diverse specificities.

### 2.4 Scn does not bind pyochelin (PCH)

**Fig. 7:**
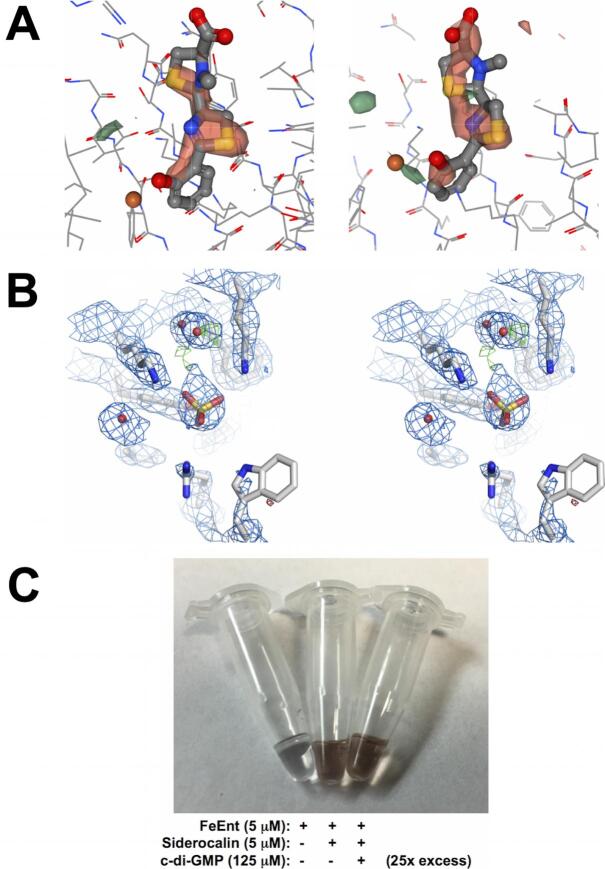
Unmasking red-herring Scn ligands. (A) Two views show the F_obs_-F_calc_ difference electron density map around the PCH ligand as built in 3U03.pdb, contoured at −3σ (red) and +3σ (green), using the NGL viewer through the PDB website (Berman et al., 2000, Rose et al., 2018). (B) A stereoview shows the 2F_obs_-F_calc_ (contoured at 1σ, blue) and F_obs_-F_calc_ difference (contoured at −3σ, red, and +3σ, green) electron density maps in the Scn calyx in the re-refinement of 3U03.pdb. W79, at the bottom right, is disordered in this structure. Compare the modeled sulfate ion position with Fig. 3E. (C) The results of the Fe-ENT/c-di-GMP competition experiment are shown in a photograph of the ultrafilter retentates. (For interpretation of the references to color in this figure legend, the reader is referred to the web version of this article.)

*Pseudomonas aeruginosa* is a Gram-negative γ-proteobacterium which can cause acute and chronic infections ranging from septicemia, urinary infections, wound colonization, and chronic lung colonization in cystic fibrosis patients (Cornelis and Dingemans, 2013). *P. aeruginosa* produces two chemically-distinct siderophores, pyoverdine (PVD) and PCH, with relatively high or low affinities for iron, respectively. We had previously shown that Scn does not appreciably bind to PVD or PCH in qualitative binding assays (Holmes et al., 2005), and does not arrest the growth of *P. aeruginosa in vitro* (Correnti et al., 2011), suggesting that Scn does not efficiently sequester one or both siderophores. The quite large PVD structure would also not be expected to fit within the Scn calyx, nor would the structure of PCH-chelated iron be expected to fit within the key binding pocket, due to tight steric constraints around the iron center imposed by K125 and K134. A preliminary crystal structure of a Scn/PCH complex (3U03.pdb) was deposited in the Protein Databank (PDB, (Berman et al., 2000)) by a structural genomics consortium. However, examination of this structure revealed a number of concerns. First, using the deposited diffraction data, it was readily apparent that the PCH ligand as modeled sat in negative electron density in difference Fourier syntheses (Fig. 7A). Second, this particular crystal form of Scn usually contains three molecules in the asymmetric unit, though only two had been modeled in the deposited structure. Using the deposited diffraction data, we re-refined the structure (Table 2), successfully placing the third Scn molecule and improving refinement statistics: R_free_/R_work_ values decreased from 0.303/0.269 (3U03.pdb) to 0.253/0.235 (Table 2). Calyx electron density features in the re-refined structure (Fig. 7B) were inconsistent with bound Fe-PCH but were consistent with a bound sulfate ion comparable to those observed in *apo*-Scn structures crystallized from ammonium sulfate, and in the phenylurea complex. We concluded that Scn does not bind PCH, at least under these crystallization conditions, in concordance with prior results.

### 2.5 Scn does not bind norepinephrine (NE)

Though hexadentate catecholate siderophores have much higher complexation constants, bidentate catecholamine hormones (e.g. norepinephrine, NE) are capable of chelating iron in 3:1 complexes, and can serve to promote bacterial growth, at least *in vitro* (O'Donnell et al., 2006). Building on the demonstrated recognition degeneracy of Scn/siderophore recognition, Miethke and Skerra reported that ferric NE complexes bound tightly to Scn, using an FQ binding assay, and could be co-crystallized with Scn, yielding deep red colored crystals (Miethke and Skerra, 2010). However, no co-crystal structure was or has been subsequently reported, and the observed crystal habit was distinct from the typical tetragonal forms observed for Scn (e.g., Fig. 6B, D, and F). Based on the specificity rules for Scn outlined above, we would have predicted that 3,4-CAM-type siderophores, like NE, would not bind, due to the tight steric constraints imposed by the rigid Scn calyx, particularly by Pocket #1, and we had previously demonstrated that simple 3,4-CAM siderophores, like 3,4-DHBA, do not bind to Scn (Correnti et al., 2012). In order to resolve this discrepancy, we undertook to directly determine the Fe-NE/Scn co-crystal structure. However, difference Fourier syntheses calculated from diffraction data collected from tetragonal crystals grown in the presence of stoichiometric 3:1 NE:iron mixtures (d_min_ = 2.4 Å, Table 2) did not show electron density features assignable to iron or ligand atoms, only ordered solvent molecules and sulfate ions. Given that the three calyx pockets have different tolerances for ligand elaboration, we also tried co-crystallizing Scn with 2:1:1 2,3-DHBA:NE:iron complexes, under the assumption that NE might be tolerated in one or another calyx pocket in a mixed chelated complex. However, difference Fourier syntheses calculated from these data (d_min_ = 2.25 Å, Table 2) did not show difference features distinct from the previously-determined Scn:2,3-DHBA:iron complex structure (Correnti et al., 2012). Since neither structure was deemed useful or informative, full structure refinements were not completed, and they have not been deposited. Based on these results, we concluded that, consistent with our developed recognition rules and prior binding studies, 3,4-CAM siderophores, like NE, are not physiologically-relevant ligands for Scn. Indeed, selective binding of 2,3-CAMs over 3,4-CAMs and more highly substituted derivatives *in vivo* would be advantageous for the Scn antibacterial defense.

### 2.6 c-di-GMP does not efficiently compete with ENT for binding to Scn

Li and coworkers reported that c-di-GMP could compete with bacterial ferric siderophores for Scn binding, alleviating Scn blockade of bacterial iron acquisition during infection (Li et al., 2015). This conclusion was based on a computational inverse docking screen, isothermal titration calorimetry (ITC) binding assays, and bacterial growth assays. However, c-di-GMP is not particularly complementary in shape to the Scn calyx, and lacks elements crucial for calyx binding, though its net negative charge does complement the overall positive charge of the calyx. Also, the reported *micromolar K_D_* by ITC for the Scn/c-di-GMP interaction was orders-of-magnitude weaker than the sub-*nanomolar K_D_*s of Scn for ferric complexes of bacterial CAM-type siderophores previously determined by FQ (Abergel et al., 2006a, Abergel et al., 2006b, Goetz et al., 2002, Hoette et al., 2008, Miethke and Skerra, 2010). [We noted that Li and coworkers also reported a discrepant *micromolar K_D_* for the Scn/Fe-ENT interaction by ITC.] However, this conundrum was easily resolved by a simple mixing experiment not previously performed (Fig. 7C). Colorless *apo*-Scn protein was premixed with a 25-fold molar excess of colorless c-di-GMP, at a concentration well above the reported micromolar *K_D_*, and allowed to equilibrate. Fe-ENT, which is colored a deep red, was then added at an equimolar ratio to Scn. When the protein was washed in an ultrafilter, the telltale Scn/Fe-Ent complex was formed and retained even after preincubation with excess c-di-GMP, indicating that c-di-GMP binding is too weak to impede binding of ferric siderophores to Scn. We speculate that an observed micromolar affinity constant could easily be the product of a non-specific electrostatic interaction and is likely not physiologically-relevant.

### 2.7 Contrasting siderophore recognition by mammals (Scn) and bacteria

Siderophores enable bacterial acquisition of essential iron, which Scn functionally competes with *in vivo*, so contrasting recognition mechanisms and specificities is needed to fully understand this physiological contest for iron. Gram-negative bacteria retrieve ferric siderophores from the extracellular milieu through specific uptake pathways comprising outer membrane receptors, PBPs, and inner membrane ATP‐binding cassette (ABC) transporters (Schalk and Guillon, 2013). Crystal structures of the Fe-Ent-specific outer membrane receptors from *P. aeruginosa* (PfeA, 5M9B.pdb, *to be published*) and *Escherichia coli* (FepA, 1FEP.pdb (Buchanan et al., 1999)), as well as solution structures of the *E. coli* Fe-Ent-specific PBP FepB (2M6L.pdb (Chu et al., 2014)), have been determined previously, but, unfortunately, do not provide details of ligand recognition, as the ligand was either not included, or the binding sites were disordered.

**Fig. 8:**
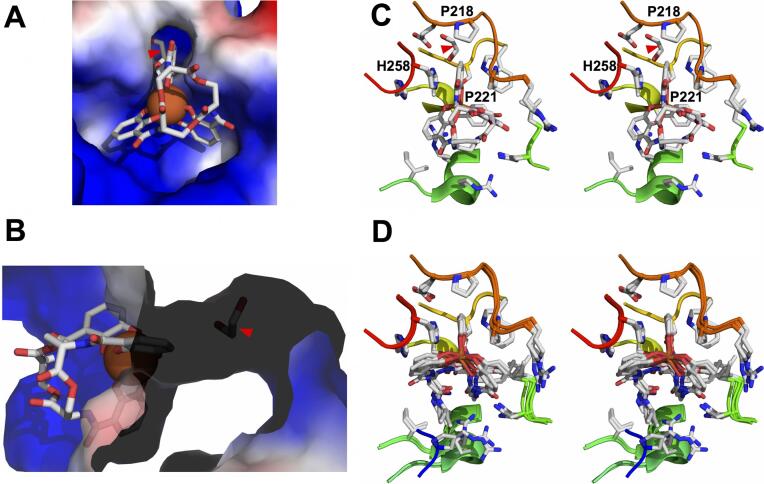
Contrasting Scn and bacterial recognition of ENT: esterase PfeE. (A) A view down into the co-crystal structure (6GI1.pdb) of the inactivated *P. aeruginosa* PfeE(S157A) ENT esterase (Perraud et al., 2018), showing binding of Fe-ENT. The protein is shown as a molecular surface, colored by electrostatic charge, ENT is shown in a licorice-stick representation, colored by atom type, and the iron atom is shown as an orange sphere. The most constrained pocket in the binding site is at top, and a co-crystallizing ethylene glycol molecule is highlighted with a red arrow. (B) A view rotated by 90° around the vertical axis from that in (A), with the molecular surface rendered semi-transparent, showing the tunnel underlying the ligand and the entrapped ethylene glycol molecule (red arrow). (C) A stereoview of the superposition of the binding sites of molecule B from 6GI1.pdb and molecule B from 6GI2.pdb shows the outwards-pointing orientation of Fe-ENT or the linearized, nonhydrolyzable ENT analog. The orientation is close to that in (A). The ligands and side-chains of close-approaching residues are shown in a licorice-stick representation, and segments of the protein backbone are shown in a cartoon representation, colored from blue-to-red, N- to C-terminus. (D) A stereoview of the superposition of the binding sites of molecule A from 6GI1.pdb, molecule A from 6GI2.pdb, and both molecules in 6GI5.pdb shows the inwards-pointing, substrate orientation of Fe-ENT or the linearized, nonhydrolyzable ENT analog. The frame is styled and oriented as in (C). (For interpretation of the references to color in this figure legend, the reader is referred to the web version of this article.)

However, recent crystal structures of the *P. aeruginosa* PfeE ENT esterase (Perraud et al., 2018) detailed Gram-negative bacterial recognition of ENT, though enzymatic binding and recognition, optimized for efficient catalysis, may have different constraints than binding for transport or sequestration. The PfeE binding site for ENT is strikingly similar in overall shape to the Scn calyx, despite completely distinct folds, and is comprised of three pockets, with a large part of bound ENT comparably exposed to solvent (Fig. 8A and B). One pocket is sterically more constrained, tightly bracketing one CAM substituent. This pocket also has a tunnel extending from underneath ENT, out through the backside of the protein, unfilled by any ENT substituent – though an ethylene glycol molecule was observed wedged in the tunnel in the crystal structure. Like Scn, the other two pockets tolerate a larger range of ligand conformers (Fig. 8C and D). While the binding site is electropositive overall, echoing Scn, the tightly-constrained pocket uses distinct bonding: the CAM moiety is bracketed by *en face* stacking from the side-chains of proline (P221) and histidine (H258) residues, and a T interaction from another proline side-chain (P218). This pocket likely corresponds to the key Pocket #1 in Scn, as a lone DHBA-serine degradation product was retained here in one view of the structure. [An inactive mutant of PfeE was co-crystallized with ENT and a linearized, non-hydrolyzable ENT analog, and the active, native enzyme was co-crystallized with the analog.] Inactivated PfeE bound ENT in an orientation echoing that of Scn, with the triserine backbone pointing outwards towards solvent (Fig. 8C). However, native PfeE bound the linearized, non-hydrolyzable ENT analog in the opposite orientation, with the triserine backbone pointing inwards, shielded from solvent (Fig. 8D). PfeE utilizes a serine catalytic triad to hydrolyze ENT lactone linkages, which has no counterpart in the Scn calyx, drawing a significant distinction. Also, the inward-pointing ligand orientation (Fig. 8D) represents the catalytically active complex. The biological relevance of the outward-pointing orientation, comparable to Scn but observed only for catalytically inactive PfeE, is unclear, and likely reflects that enzyme binding is optimized for the transition state, and not the substrate. Therefore, despite some superficial similarities, PfeE and Scn ENT recognition mechanisms appear quite distinct.

**Fig. 9:**
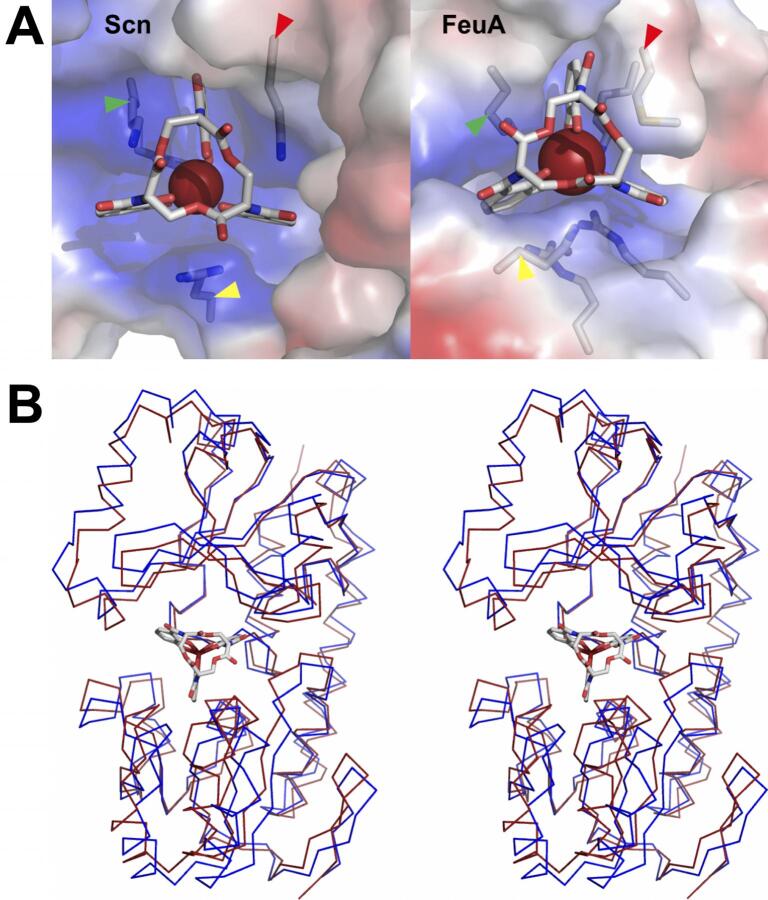
Contrasting Scn and bacterial recognition of ENT: SBP FeuA. (A) Side-by-side views compare and contrast the structures of the human Scn/Fe-ENT (1L6M.pdb; (Goetz et al., 2002)) and the *B. subtilis* FeuA/Fe-ENT complexes (2XUZ.pdb; (Peuckert et al., 2011)). Proteins are shown as semi-transparent molecular surfaces, colored by electrostatic charge, with the side-chains of key ligand-interacting residues shown in a licorice-stick representation. Side-chains are marked by colored arrows to indicate rough spatial equivalence: Scn K125/FeuA K105: green; Scn K134/FeuA M85: red; and Scn R81/FeuA Q215: yellow. (B) A stereoview of the superposition of the Fe-ENT-bound (red) and unbound (blue) structures of FeuA, rendered as Cα backbone ribbons, highlights the ligand-induced conformational change. Bound Fe-Ent is shown in a licorice-stick representation. (For interpretation of the references to color in this figure legend, the reader is referred to the web version of this article.)

Gram-positive bacteria alternately retrieve ferric siderophores through siderophore-specific SBPs and associated ABC-type transporters (Chu et al., 2010, Schalk and Guillon, 2013). The crystal structure of the *Bacillus subtilis* SBP FeuA/Fe-ENT complex (2XUZ.pdb (Peuckert et al., 2011)) provided an excellent contrast with Scn-mediated recognition (Fig. 9A). While Fe-ENT is relatively exposed in both complexes, and both binding sites are electropositive, few other similarities were noted. The overall shape of the binding sites is quite distinct, as are the identities of CAM-intercalating residues. Rather than relying on cation-π interactions as in Scn, FeuA brackets the CAM substituent in the tightest pocket with the aliphatic portion of a lysine (K105) side-chain and a methionine (M85) side-chain. As has been frequently observed in PBPs and SBPs, Fe-ENT binding is accompanied by significant protein conformational changes (Fig. 9B), quite unlike Scn. This recognition mechanism also dramatically contrasts with Scn in that FeuA displays only limited cross-recognition of related ligands, even making distinctions at the level of stereoconfiguration, which is alternately plastic in the Scn calyx (e.g., Fig. 4C). Functionally, bacterial siderophore receptors and binding proteins reasonably appear driven by the need for *specificity*, while Scn-mediated anti-bacterial responses are enhanced by recognition *breadth*.

**Fig. 10:**
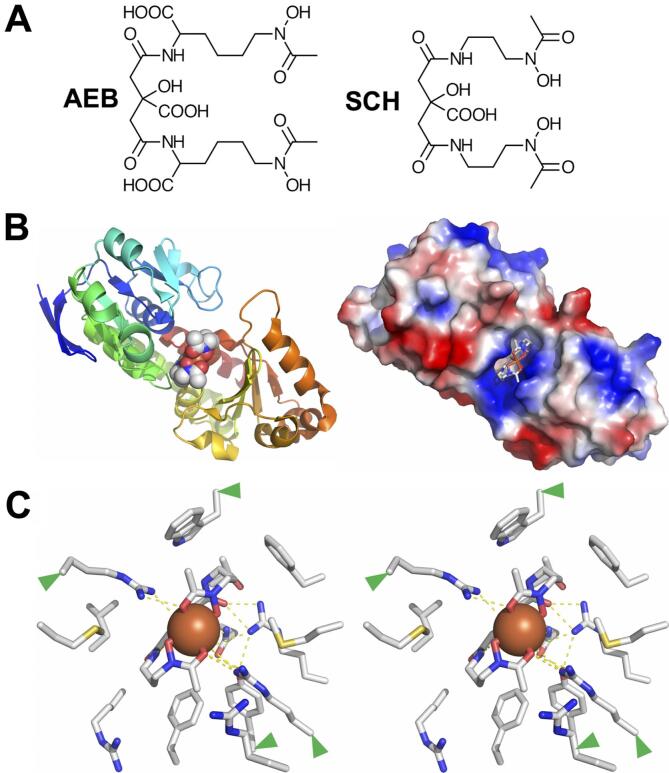
The structure of the YfiY/SCH complex. (A) The chemical structures of AEB (left) and SCH (right) are compared. (B) A cartoon ribbon representation of the *B. cereus* YfiY/Fe-SCH complex structure is shown at left, colored from blue-to-red, N- to C-terminus. The bound ligand is shown in a CPK representation, colored by atom type. A molecular surface representation of the YfiY/Fe-SCH complex structure is shown at right, colored by electrostatic potential. The bound ligand is shown in a licorice-stick representation. (C) A stereoview of the bound ligand in the YfiY/Fe-SCH complex structure is surrounded by the side-chains of neighboring residues in the protein. Hydrogen bonds are indicated by dashed lines. Residues conserved between *B. cereus* YfiY and *S. aureus* SirA are marked with green arrows. (For interpretation of the references to color in this figure legend, the reader is referred to the web version of this article.)

*B. cereus* produces the 2,3-CAM-based siderophore bacillibactin and the citrate- and 3,4-CAM-based siderophore petrobactin, but can also acquire iron through the facultative use of exosiderophores, including ENT, ferrichromes, and SCH (Zawadzka et al., 2009). SCH is a mixed-type α-hydroxy acid/hydroxamate siderophore, chemically similar to AEB, a virulence-associated siderophore that enables evasion of Scn-mediated bacteriostatic effects (Fig. 10A) (Flo et al., 2004, Goetz et al., 2002, Sheldon and Heinrichs, 2015). To contrast bacterial recognition of SCH or AEB with the inability of Scn to bind this type of siderophore, we determined the crystal structure of *B. cereus* YfiY in complex with Fe-SCH (d_min_ = 1.55 Å, Table 3, Fig. 10B and C). Overall, YfiY displays the typical SBP fold (Berntsson et al., 2010, Scheepers et al., 2016), a bilobate structure with the ligand binding site sitting at the domain juncture. Based on Dali server searches (Holm and Laakso, 2016), YfiY is most structurally similar to the ligand-bound structures of the *Staphylococcus aureus* staphyloferrin-specific SBPs SirA (3MWF.pdb, superposition rmsd = 1.8 Å; 40% sequence identity (Grigg et al., 2010a)) and HtsA (3LI2.pdb, superposition rmsd = 2.1 Å; 30% sequence identity (Grigg et al., 2010b)). [Staphyloferrin is a pentacarboxylic acid derivative of D-ornithine.] Like PBPs, SBPs typically undergo domain closure in response to ligand binding, and YfiY would be predicted to do so as well, as the structural similarity dropped when compared to the ligand-free structure of SirA (3MWG.pdb, superposition rmsd = 2.2 Å (Grigg et al., 2010a)). The SCH binding site is a deep pocket between the N- and C- terminal lobes (Fig. 10B) lined by polar amino acid side-chains from R91, T110, and R112 from the N-terminal lobe and R162, R169, R200, and N261 from the C-terminal lobe, and hydrophobic amino acid side-chains from W47 and M90, from N-terminal lobe and M164, Y171, F197 and F221 from the C-terminal lobe. Four arginine residues (91, 112, 162 and 169), Y171 and N261 make direct hydrogen bonds to Fe-SCH (Fig. 10C). Additional water-mediated hydrogen bonds further stabilize binding. This SCH recognition mechanism is wholly distinct from ENT recognition mechanisms discussed above, highlighting the range of evolutionary solutions to moving iron.

**Table 3:** Crystallographic data collection and refinement statistics for *B. cereus* YfiY.

Protein	*B. cereus* YfiY
Ligand	Fe-SCH
Accession Code	3TNY
Space group	P2_1_

Cell dimensions	
a, b, c (Å)	57.796, 47.328, 64.981
α, β, γ (°)	90, 112.88, 90
Resolution (Å)	50.0–1.55 (1.61–1.55)
*R*_merge_	0.072 (0.12)
I/σ(I)	11.8 (3.27)
Completeness (%)	99.5 (94.7)
Redundancy	7.3 (4.8)
*R*_work_/*R*_free_	0.172/0.189

*No. atoms*	
Protein	2170
Heterogen	30
Waters	374
Wilson B (Å^2^)	18.9
Average B (isotropic, Å^2^)	21.1

*R.m.s. deviations*	
Bond lengths (Å)	0.006
Bond angles (°)	1.11

Values in parentheses are for the highest-resolution shell.

## 3 Conclusions

Scn displays exceptional structural rigidity, manifested by its near-identical structure across multiple crystal structures, bound to a series of distinct ligands, tolerating multiple mutations. Scn also displays a multiplexed recognition mechanism for distinct families of natural and synthetic chelators and degradation products, free or bound to any of a series of metals. Multiplexed Scn recognition, however, is not boundless. A series of proposed candidate ligands, some functionally related to *bona fide* ligands, was demonstrated not to bind with appreciable affinities. Recognition breadth, maximizing the reach of the Scn antibacterial defense, was achieved by the unique evolution of a binding site focusing on key, shared ligand substituents in an otherwise permissive calyx, utilizing a flexible bonding network. The uniqueness of the Scn recognition mechanism was demonstrated by the distinct specificity mechanisms alternately used by bacterial transporters and enzymes.

## 4 Materials and methods

*Protein biochemistry and crystallography*: Mutations were made as previously described (Abergel et al., 2008, Goetz et al., 2002). All clones were confirmed by DNA sequencing. Scn proteins were expressed and purified as previously described (Abergel et al., 2008, Bundgaard et al., 1994, Goetz et al., 2002). YfiY was expressed and purified as previously described [49]. All crystals were grown using hanging drop vapor diffusion at room temperature. With the exception of Scn(C87S/K125A) with Fe-ENT, and Scn(C87S/K125A/K134A) with Fe-ENT, Scn was isomorphously co-crystallized in the presence of ligands as described previously (Goetz et al., 2002, Holmes et al., 2005). Scn(C87S/K125A) was isomorphously co-crystallized from 0.2 M (NH_4_)_2_SO_4_ and 30% w/w polyethylene glycol 4000, and Scn(C87S/K125A/K134A) was isomorphously co-crystallized in 0.2 M (NH_4_)_2_SO_4_, 25% w/w polyethylene glycol 4000, and 15% v/v glycerol. YfiY was crystallized at a concentration of 10–20 mg/mL from 0.1 M HEPES (pH = 7.0) and 30% w/w Jeffamine ED-2001 at ambient temperature. Crystals were cryoprotected by adding glycerol to 15% v/v, and flash cooled to −170 °C. All data sets were collected in-house using CuKα radiation, or at the Advanced Light Source, beamlines 5.0.1 or 5.0.2, at a wavelength of 1.0 Å. Data sets were indexed and scaled using the HKL2000 software package (Otwinowski and Minor, 1997). Reflections used in calculating R_free_ were matched to the same R_free_ data set from the initial wild-type Scn structure (1L6M.pdb). Initial Scn structure phases were calculated from the 1L6M.pdb structure and optimized by rigid-body refinement using REFMAC5 (Murshudov et al., 1997), or by molecular replacement using 1L6M.pdb as the search model with PHASER (McCoy et al., 2007). Modeling and additional refinement was performed using Coot and REFMAC5 (Emsley and Cowtan, 2004, Murshudov et al., 1997). Molecular images were generated with MacPyMOL (DeLano, 2002, Schrodinger, 2010). FQ binding analyses were performed as previously described (Abergel et al., 2006a, Abergel et al., 2006b, Goetz et al., 2002, Hoette et al., 2008, Miethke and Skerra, 2010).

*Synthesis of bisHA-CAM* (see Fig. 3A): Compound (**2**) was prepared from Compound (**1**) by a modification of established procedures (Hu and Miller, 1994): Compound (**1**) was dissolved in CH_2_Cl_2_ and H_2_O, trifluoracetic acid was added, and the solution was stirred. Solvents were removed *in vacuo* and the residue was resuspended in saturated NaHCO_3_. The aqueous phase was extracted with CH_2_Cl_2_, and the organic phase dried over Na_2_SO_4_ and evaporated, yielding Compound (**2**). Compound (**3**) was prepared from Compound (**2**) as previously described (Hu and Miller, 1994). Compound (**3**) was dissolved in methanol, palladium-on-carbon catalyst was added, and the mixture was hydrogenated for 1.5 h under ambient conditions. The catalyst was filtered, and the solvent was evaporated to yield Compound (**4**). Compound (**5**) was prepared from Compound (**3**) as previously described (Hu and Miller, 1994). HATU was added to Compound (**4**) and Compound (**5**) in DMAA solution, which was basified to pH = 9 with triethanolamine and stirred for 15 h. The solvent was removed *in vacuo* and the residue dissolved in CH_2_Cl_2_ and washed with 0.1 M HCl. The organic phase was condensed and applied to a silica column (Merck silica gel, 40–7 mesh). The product was eluted with CH_2_Cl_2_/methanol (100:0 to 90:10) to yield Compound (**6**). Compound (**6**) was dissolved in methanol, palladium-on-carbon catalyst was added, and the mixture was hydrogenated for 3 h under ambient conditions. The catalyst was filtered, and the solvent was evaporated to yield Compound (**7**). Purities were determined step-by-step by ^1^H NMR spectroscopy at room temperature on Bruker AVB-300/400 or DRX-500 FT spectrometers.

## 5 Accession numbers

Coordinates and structure factors have been deposited in the Protein Data Bank with accession numbers 3CMP, 3HWD, 3HWE, 3HWF, 3HWG, 3I0A, 3K3L, 3T1D, 3TF6, 6O5D, and 3TNY.

## Declaration of Competing Interest

The authors declare no conflict of interest with respect to this publication.
